# Hydrolyzed rice formula for dietary management of infants with cow's milk allergy^☆^

**DOI:** 10.1016/j.waojou.2022.100717

**Published:** 2022-11-17

**Authors:** Alessandro Fiocchi, Josefa Barrio-Torres, Christophe Dupont, Helen Evans Howells, Raanan Shamir, Carina Venter, Rosan Meyer

**Affiliations:** a Director of Allergy, Pediatric Hospital Bambino Gesù IRCCS, Rome, Italy; b Pediatric Gastroenterologist, Hospital Universitario de Fuenlabrada, Madrid, Spain; c Pediatric Gastroenterology Department, Paris-Descartes University, Paris, France; d Marcel Sembat Clinic, Boulogne, France; e Royal Bournemouth Hospital, Frailty Visiting GP for Bournemouth East Collaborative, Bournemouth, UK; f Institute for Gastroenterology, Nutrition and Liver Diseases, Schneider Children's Medical Center, Petach Tikva, Israel; g Sackler Faculty of Medicine, Tel Aviv University, Tel Aviv, Israel; h Allergy & Immunology, University of Colorado Denver School of Medicine, Children's Hospital Colorado, CO, USA; i Department of Paediatrics, Imperial College London, London, UK; j Department Nutrition and Dietetics, University of Winchester, UK

**Keywords:** Children, Consensus, Cow's milk allergy, Hydrolyzed protein, Infant feeding, Hydrolyzed rice formula

## Abstract

**Background:**

Allergic diseases are increasing globally and are a significant public health challenge, especially in children. Cow's milk allergy (CMA) is one of the most common food allergies in early childhood. When CMA is diagnosed and exclusive breastfeeding is not possible, current guidelines recommend extensively hydrolyzed formulas (eHF) or amino acid-based formulas (AAF), depending on the diagnosis and severity of symptoms. Hydrolyzed rice formulas (HRF) are considered an alternative to eHF and AAF where available.

**Objectives & methods:**

We aimed to understand how HRF are being used and their position in the management of CMA, and to generate consensus on indications for use. Two virtual roundtables of international healthcare experts in pediatric food allergy focused on HRF were convened in May and October 2021. Experts provided multiple perspectives due to different specialties, locations, healthcare settings, and availability of HRF. Following discussion of international CMA guidelines and HRF use, identification of challenges along the diagnosis and treatment pathway, and translation of guidelines into clinical practice, an expert consensus on the use of HRF for CMA was developed. This involved repeated voting followed by statement amendment to reach 100% agreement. This publication summarizes insights from these meetings.

**Results:**

There was 100% agreement on all 8 statements: (1) While breastmilk is the best source of nutrition for infants with CMA, when breastfeeding is not possible, a hypoallergenic formula can be used; (2) Per definition, a HRF is cow's milk protein-free; (3) A minority of infants with immunoglobulin (Ig)E-mediated CMA react to eHF due to residual cow's milk protein; (4) More infants with non-IgE-mediated CMA than IgE-mediated CMA react to eHF likely due to residual cow's milk protein; (5) When a diagnostic elimination diet is indicated, HRF can be used; (6) A HRF can be recommended as a first-line option for CMA, where available, as outlined in the Diagnosis and Rationale for Action against Cow's Milk Allergy guidelines; (7) HRFs have proven hypoallergenicity and are suitable for the dietary management of CMA; (8) HRFs have been shown to support growth in infants with CMA, similar to other hypoallergenic formulas. Participants recommend healthcare professionals take these statements as guidance to use HRF in clinical practice. Based on efficacy and growth evidence, the experts found HRF a suitable option for the dietary management of CMA.

**Conclusion:**

HRF can be recommended as a first-line alternative to cow's milk-based eHF or AAF, where available, in the dietary management of CMA.

## Background

The prevalence of allergic diseases is increasing globally and is considered a significant public health challenge, especially in children, mainly due to the ability of allergic diseases to cause severe or life-threatening reactions.^1^, ^2^, ^3^ Cow's milk allergy (CMA) is one of the most common food allergies in childhood.^4^, ^5^, ^6^ It presents with a wide range of symptoms that place a significant burden on both the child and their caregivers.^4^ Food allergy management strategies include individualized avoidance measures and identifying suitable alternatives for a nutritionally balanced diet.^2^ The World Health Organization (WHO) recommends breastmilk as the ideal source of nutrition for infants and in the cow's milk allergic infant, breastfeeding should be maintained as long as mutually desired by the child and the mother.^7^ This guidance is supported by all food allergy associations.^6^^,^^8^, ^9^, ^10^, ^11^, ^12^, ^13^, ^14^, ^15^, ^16^, ^17^, ^18^, ^19^, ^20^, ^21^, ^22^, ^23^, ^24^, ^25^, ^26^, ^27^, ^28^, ^29^, ^30^, ^31^, ^32^, ^33^, ^34^, ^35^ However, when breastfeeding is not possible, there are varying opinions about the best formula substitute for infants with CMA, which depends on availability and the local healthcare system.^5^^,^^36^

Clinical practice guidelines and position papers assist healthcare professionals (HCPs) to improve the quality of diagnosis and management of CMA by tailoring the choices for each patient as summarized in Box 1.^6^^,^^8^, ^9^, ^10^, ^11^, ^12^, ^13^, ^14^, ^15^, ^16^, ^17^, ^18^, ^19^, ^20^, ^21^, ^22^, ^23^, ^24^, ^25^, ^26^, ^27^, ^28^, ^29^, ^30^, ^31^, ^32^, ^33^, ^34^, ^35^ However, guidelines alone are not sufficient to support HCPs and there is a need for implementation strategies to be introduced into daily practice.^1^

Box 1 **National and International Cow's Milk Allergy Position Papers and Guidelines in Children**

• Australia (Australian Society of Clinical Immunology and Allergy [ASCIA]^42^)
• China (based on World Allergy Organization DRACMA guidelines and an independent group reading of DRACMA guidelines^15^^,^^20^)
• Diagnosis and Rationale for Action against Cow's Milk Allergy (DRACMA)^14^^,^^16^^,^^29^^,^^36^
• British Society for Allergy & Clinical Immunology (BSACI)^21^
• European Academy of Allergy and Clinical Immunology (EAACI)^24^^,^^26^^,^^27^
• European Society for Paediatric Gastroenterology Hepatology and Nutrition (ESPGHAN)^19^
• Finland (Finnish Allergy Programme)^28^
• France (Committee on Nutrition of the French Society of Paediatrics^10^^,^^11^)
• India (Indian Society of Pediatric Gastroenterology, Hepatology and Nutrition^23^)
• Italy (Emilia-Romagna Working Group for Paediatric Allergy and by the Emilia-Romagna Working Group for Paediatric Gastroenterology^9^ and Italian Society of Pediatric Allergy^32^)
• Japan (Japanese Society of Pediatric Allergy and Clinical Immunology [JSPACI]; Japanese Society of Allergology [JSA]^12^)
• Mexico (independent group^25^)
• The Middle East^30^
• South America^33^, ^34^, ^35^
• Spain (Spanish Society of Paediatric Gastroenterology, Hepatology, and Nutrition [SEGHNP], Spanish Association of Paediatric Primary Care [SAEPAP], Spanish Society of Extra-hospital Paediatrics and Primary Health Care [SEPEAP], Spanish Society of Paediatric Clinical Immunology, Allergy, and Asthma [SEICAP]^13^^,^^22^)
• Turkey (Turkish Society of Pediatrics)^18^
• United Kingdom (National Institute for Health and Care Excellence (NICE) Milk Allergy in Primary Care [MAP]^31^^,^^43^ and international MAP [iMAP]^6^^,^^17^)
• United States (American Academy of Allergy, Asthma, and Immunology [AAAAI]^44^ and National Institute of Allergy and Infectious Diseases [NIAID]^19^)

Alt-text: Box 1

Formulas adapted for infants with CMA, although mostly made from hydrolyzed cow's milk protein or amino acids, can be made from hydrolyzed proteins from other sources. Regardless of protein source, these formulas have to comply with relevant food regulations, be nutritionally complete to support normal growth and development in infants, and have to undergo clinical trials to support efficacy.^4^ Formulas made from hydrolyzed rice protein have been on the market in Europe since the 2000s as a nutritionally adequate and well-tolerated plant-based alternative to cow's milk protein-based extensively hydrolyzed formulas (eHF) and for the dietary management of CMA.^4^^,^^37^, ^38^, ^39^, ^40^, ^41^

This publication summarizes the presentations, discussions, and consensus from a 2021 expert roundtable series discussing the position of HRF in the dietary management of infants with CMA where breastmilk is insufficient or not available.

## Background to expert roundtable series discussing the position of HRF in the dietary management of infants with CMA

In May 2021, the first of a two-part virtual roundtable series was held for international HCPs with expertise in pediatric food allergy (pediatric gastroenterologists [n = 2], dietician [n = 1], pediatric allergist/immunologists [n = 2], and non-specialist HCP [n = 1]). Representative countries included France, Israel, Italy, Spain, the United Kingdom, and the United States. The primary goal was to provide clear direction on when and where HRF can be used for the dietary management of CMA (Box 2). The meeting was chaired by a specialist pediatric allergy dietician. The experts provided multiple perspectives due to their different specialties, locations, and healthcare settings.

Box 2 **Meeting objectives/goals**

**Table:** 


1) Understand how HRF are being used and how they are positioned in current international CMA guidelines.
2) Identify the challenges along the diagnosis and treatment pathway and explore HRF as a dietary management option.
3) Generate consensus on how CMA guidelines can help drive clinical practice change.
4) Provide clear direction on when and where HRF can be used.

CMA, cow’s milk allergy; HRF, hydrolyzed rice formulas

Alt-text: Box 2

**Fig. 1:**
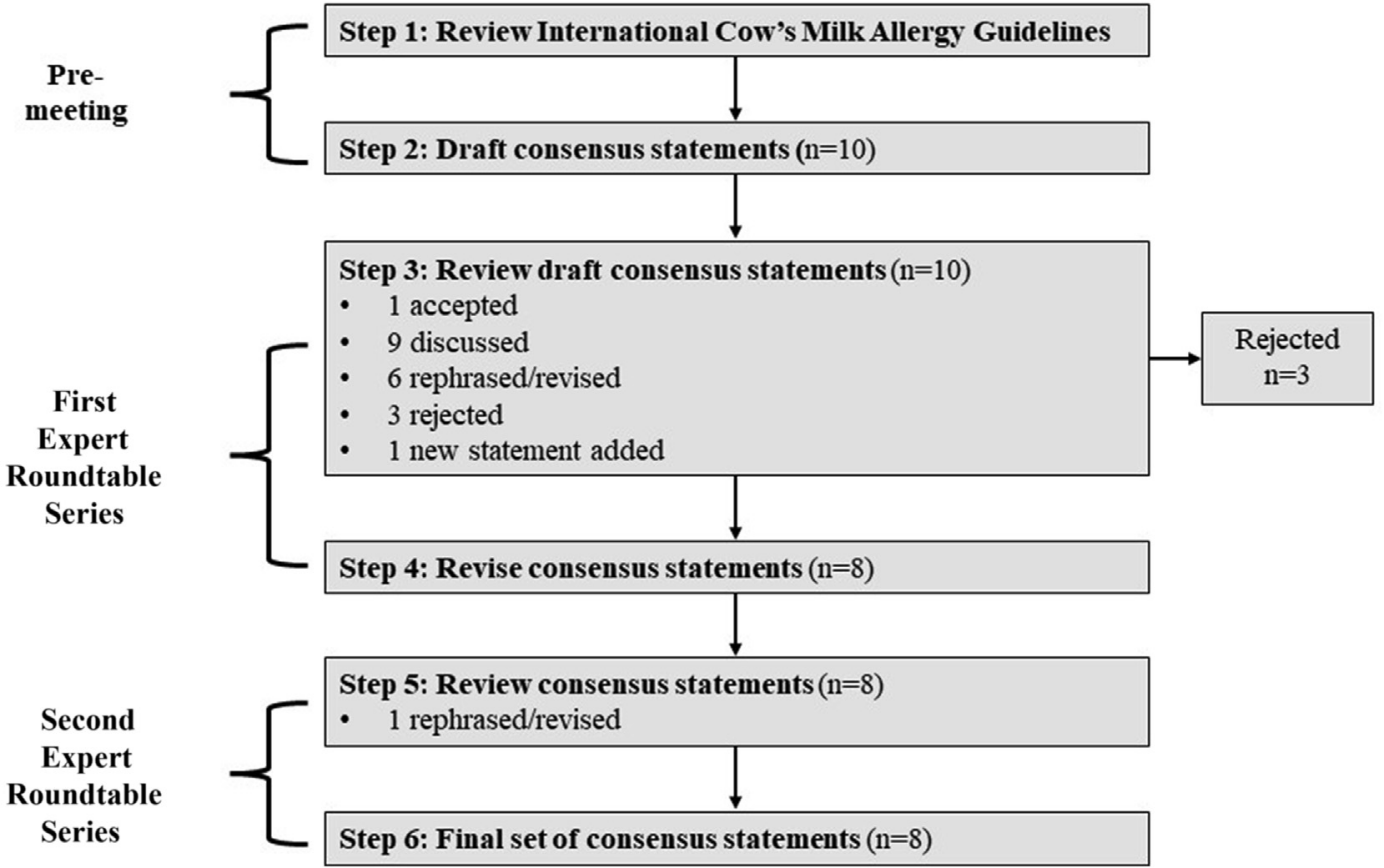
Facilitated expert roundtable discussions on consensus statements on the use of HRF for HCPs.

The first roundtable was divided into 4 sections: a) existing international CMA guidelines and how HRF are being used internationally; b) identification of the challenges on the diagnosis and treatment pathway; c) how CMA guidelines can be translated into clinical practice; and d) expert consensus on the use of HRF for CMA. The key discussion points were captured by a medical writer. Based on the available evidence from the review of international CMA guidelines and recently published studies, a series of statements, relevant to each of the 3 sections described above, were developed, and presented to the participating experts. To achieve expert consensus, voting was used to facilitate the discussion and amend the statements where 100% of agreement was not achieved. The amended statements where then circulated through e-mail to the expert group before the second roundtable, to ensure that all feedback from the first meeting was captured and statements were amended accordingly. In October 2021, the second virtual roundtable was held with the same international experts for a second-round discussion of the consensus statements, adjustment of wording where necessary, and finalization (Fig. 1).

The primary goal of this expert roundtable series was to provide clear direction on when and where HRF can be used for the dietary management of CMA. The following sections capture the main discussion points among the experts and supporting evidence from the literature.

## How HRF are being used internationally and an overview of international CMA guidelines

Current global CMA guidelines support breastfeeding, where available, as a first option for infant feeding. All experts in attendance were highly supportive of this. HRF are an established option for infants with CMA, but their use has been limited by geographical availability and HCP awareness. HRF have been used since the early 2000s in several European countries (France, Italy, and Spain) but are not widely available in other regions.^4^ HRF already account for 4.9% of all formulas used for children aged 0–3 years in France.^37^

The experts discussed how HRF are positioned in current international CMA guidelines (Table 1). Prominent guidelines such as the WAO DRACMA Guidelines^16^ from 2010, and in the process of being updated in 2022,^14^^,^^29^ and ESPGHAN^19^ from 2012 include guidance on the use of HRF in the management of CMA. Local CMA guidelines from Australia,^42^ China,^15^^,^^20^ France,^10^^,^^11^ Italy^9^^,^^32^ and Spain^13^^,^^22^ include recommendations on HRFs. Where such formulas are not available, they are not included (eg, BSACI,^21^ EAACI,^26^^,^^27^ Finland,^28^ India,^23^ Japan,^12^ Mexico,^25^ the Middle East,^30^ NIAID,^8^ South America,^34^^,^^35^ Turkey,^18^ UK NICE Map/iMAP and Primary Care,^6^^,^^31^^,^^43^ and AAAAI^44^).

**Table 1:** International CMA guidelines recommend HRF as a suitable alternative to eHF, where appropriate.^16^^,^^19^

Clinical presentation	First-line option		Second-line option	
	DRACMA 2010^16^	ESPGHAN 2012^19^	DRACMA 2010^16^	ESPGHAN 2012^19^
Anaphylaxis	AAF	AAF	eHF/HRF^b^	–
Immediate gastrointestinal allergy	eHF/HRF^b^	eHF in <12 mo of age eHF/SF in >12 mo of age∗	AAF	AAF in <6 mo of age eHF/SF in >6 mo of age HRF^d^^,^∗
Atopic dermatitis (eczema)	eHF/HRF^b^	eHF in <12 mo of age eHF/SF in >12 mo of age∗	SF^a^/AAF^c^	AAF in <6 mo of age eHF/SF in >6 mo of age HRF^d^^,^∗
Food protein-induced enterocolitis syndrome/severe enteropathy indicated by hypoproteinemia and failure to thrive	AAF^e^	eHF^e^	eHF if child refuses AAF	–
Allergic eosinophilic esophagitis	AAF	AAF	–	–
Respiratory symptoms (e.g., rhinitis and asthma)	eHF/HRF^b^	eHF∗	SF^a^/AAF^c^	AAF/HRF^d^^,^∗
Skin symptoms (e.g., acute urticaria, angioedema)	eHF/HRF^b^	eHF in <12 mo of age eHF/SF in >12 mo of age∗	SF^a^/AAF^c^	AAF in <6 mo of age eHF/SF in >6 mo of age HRF^d^^,^∗
Gastrointestinal symptoms (e.g., constipation, gastroesophageal reflux disease, severe irritability [colic])	eHF/HRF^b^	eHF in <12 mo of age eHF/SF in >12 mo of age∗	AAF	AAF in <6 mo of age eHF/SF in >6 mo of age HRF^d^^,^∗
Food protein-induced enteropathy	eHF/HRF^b^	eHF in <12 mo of age eHF/SF in >12 mo of age^f^^,^∗	AAF	AAF in <6 mo of age eHF/SF in >6 mo of age HRF^d^^,^∗

AAF: amino acid-based formula; DRACMA: Diagnosis and Rationale for Action against Cow's Milk Allergy; eHF: extensively hydrolyzed formula; ESPGHAN: European Society of Pediatric Gastroenterology Hepatology and Nutrition: FPIES: Acute food protein-induced enterocolitis syndrome; HRF: hydrolyzed rice formulas; SF: soy formula.

∗The options are an interpretation of the food allergy guidelines as the symptoms/conditions mentioned were not specified; use of a hypoallergenic formula was generally recommended (eHF or if allergic symptoms persist, AAF).

a Only in infants aged >6 months.

b When available, HRF can substitute for eHF.

c If the infant/child is at risk of sensitization to soy proteins and caregivers can afford them, AAF will be recommended instead of SF.

d May be considered in selected infants, which are either refusing or not tolerating an eHF based on cow's milk protein, or in vegan families.

e The most recent FPIES food allergy guidelines recommend both eHF and AAF for the nutritional management of FPIES.^24^

f If failure to thrive is present, an AAF may need to be considered as first-line feed^24^

As use of HRF becomes more widespread in many other regions such as North Africa, the Middle East, and Latin America, it is important that there is current and consistent guidance from experts on when and where to use HRF, to increase confidence, and provide evidence-based recommendations on their safety and efficacy.

## Identifying challenges along the diagnosis and treatment pathway

Despite the availability of international CMA guidelines, significant delays exist in the journey from diagnosis to optimal management of CMA. There remain notable differences between parents' and HCPs' beliefs on presentation and management of this condition, which has clinical implications for the child and the family alike.^45^ Whilst some CMA guidelines^6^^,^^17^ have tried to classify CMA based on severity, it is difficult to define *“mild-to-moderate”* CMA as it is not only contingent on the symptoms of the patient but must also take account of quality of life and the economic burden of the disease on the patient, family, and healthcare system. Overdiagnosis of CMA exists, which in turn could lead to overuse of HRF (and other hypoallergenic formulas).^17^^,^^46^^,^^47^ Guideline-defined symptoms of non-IgE-mediated CMA are still very common in infants.^47^ Appropriate diagnostic re-introduction of cow's milk formula must be performed to confirm a non-IgE-mediated CMA to ensure formula usage is appropriate as per current CMA guidelines.^16^^,^^19^

All HCPs from primary to tertiary care need to be involved in the decision-making process to introduce a new formula; however, many challenges still remain on moving from guidance to implementation. Experts highlighted that misdiagnosis and inappropriate use of hypoallergenic formulas still occurs, in addition to formula switching before a 2–4-week elimination period has been completed. Differences in regional healthcare systems was also highlighted as a contributing factor. Managing parental expectations can also be challenging for HCPs and subsequently impact formula switching (eg, not allowing sufficient time for the formula to have an effect, leading to formula switches in less than 2 weeks, which is contrary to current CMA guidelines).^16^^,^^19^

There was agreement that a comprehensive diagnosis and proper diagnostic procedures could help to ensure hypoallergenic formulas are prescribed more appropriately in non-breastfed infants, but there are opportunities for improvement, particularly among non-specialist HCPs. Further, HCPs also need to be involved in the development and execution of joint food allergy guidelines in the management of CMA, to help advise and set expectations appropriately for parents to prevent premature switching. It needs to be made clear that when trialing a formula, improvement of symptoms will take time.^13^

In countries such as France, where HRF are widely available, it is common for HCPs to switch from a cow's milk formula to a hypoallergenic formula if the current formula does not work.^4^ In fact, in France HRFs account for 4.9% in volume of all formulas for children aged 0–3 years.^37^ The symptoms and clinical presentation of each patient should be considered, and an individualized treatment plan should be put in place. This may also be further determined by healthcare systems, including reimbursement, medical insurance, economic circumstances, and the child's age.

Plant-based drinks cannot be considered equivalent to infant formulas; they are not nutritionally complete and are, therefore, unsuitable to support the growth and development of children.^48^^,^^49^ Plant-based drinks must not be used in children under 6 months, but can be added to food/in cooking, not as the main drink between 6 months and 1 year of age. Only fortified plant-based drinks can be used in children after 1 year of age if the following criteria are met by the child: eats a varied solid-food diet with a variety of foods from each food group; gets at least two-thirds of their energy from the varied solid-food diet; consumes no more than 16 ounces/500 mL of milk substitute per day (includes breastmilk, formula, and other dairy substitutes, e.g., yogurt); eats age-appropriate textures; and gets sufficient protein, fat, and micronutrients in the diet from solid foods and the available milk substitute; no feeding difficulties that may reduce food variety; no known micronutrient deficiencies; and no religious/cultural dietary requirements that reduces the variety of foods consumed.^48^^,^^49^

## Translating CMA guidelines into clinical practice

While specialists mainly follow international CMA guidelines, non-specialist HCPs are more likely to use local CMA guidelines and/or guidance from professional associations as it more practical. It is therefore critical that international CMA guidelines^16^^,^^19^ provide updated information on HRF to support secondary and tertiary care CMA guidelines for local primary HCPs.

Peer-to-peer support, particularly confidence among physicians with previous experience of HRF and among secondary physicians, is critical to support the widespread acceptance of HRF. However, positioning of HRF in the CMA treatment landscape will take time. In addition, more clinical trials will be needed so that physicians feel confident in the robust body of evidence available. The most compelling attributes of HRF differ based on the physician's area of specialty and/or clinical caseload. CMA guidelines have the potential to provide clinical recommendations on the use of all hypoallergenic formulas, not only HRF.

## Expert consensus recommendations on the use of HRF for CMA

Draft consensus statements were developed based on the most recent international CMA guidelines and recommendations. Relevant background materials were distributed to all attendees prior to the first roundtable (a presentation describing the clinical evidence on HRF and two review articles by Dupont and Bocquet^4^^,^^37^).

At the first roundtable meeting, 10 draft consensus statements, based on current published evidence, were presented to the experts. All statements were significantly edited/co-created by the HCPs in attendance – stimulus was presented, and the HCPs crafted appropriate statements (rejected n = 3; new statement n = 1). At the end of the first roundtable there were 8 revised consensus statements (Fig. 1). At the second roundtable, one statement was revised, and the final set of statements was confirmed. See Table 2 for the final, agreed-upon consensus statements on the use of HRF in infants with CMA. The domains of the 8 consensus statements can be broadly categorized into the use of hypoallergenic formulas when breastmilk is not available or insufficient, eHF in IgE and non-IgE mediated allergy, the position of HRF in the management of CMA, and safety and growth of these formulas. There was consensus that immunoglobulin E (IgE)-mediated and non-IgE-mediated CMA should be clearly distinguished as separate entities and treated accordingly (statements 3 and 4). During discussions on the statements, concerns over natural inorganic arsenic content in rice milk were highlighted as being specific to the United Kingdom, as there is clear guidance on these products not to be used as a substitute for milk before the age of 5 years;^50^ however, this is not relevant for HRF. A recent study found that inorganic arsenic levels in HRF are within the safety range as stipulated in European Food Safety/World Health Organization regulations.^51^ Ultimately, there was strong belief among the experts in the evidence-based benefits of HRF as a first-line option, where available, for the dietary management of CMA.

**Table 2:** Consensus statements on the use of HRF in infants with CMA.

No.	Statement
1	While breastmilk is the best source of nutrition for infants with CMA, when breastfeeding is not possible, a hypoallergenic formula can be used
2	Per definition, a hydrolyzed rice formula is cow's milk protein-free
3	A minority of infants with IgE-mediated CMA react to extensively hydrolyzed formulas due to residual cow's milk protein
4	More infants with non-IgE-mediated CMA than IgE-mediated CMA react to extensively hydrolyzed formulas likely due to residual cow's milk protein
5	When a diagnostic elimination diet is indicated, hydrolyzed rice formula∗ can be used
6	A hydrolyzed rice formula can be recommended as a first-line option for CMA, where available, as outlined in the DRACMA guidelines
7	Hydrolyzed rice formulas have proven hypoallergenicity and are suitable for the dietary management of CMA
8	Hydrolyzed rice formulas have been shown to support growth in infants with CMA, similar to other hypoallergenic formulas

CMA: cow's milk allergy: DRACMA, Diagnosis and Rationale for Action against Cow's Milk Allergy; Ig: immunoglobulin. ∗Approved for use by the European Union and the US Food and Drug Administration

## Conclusions

In conclusion, experts support the evidence-based research of HRF as a suitable option for the dietary management of CMA when breastmilk is insufficient or unavailable, as presented in the final consensus statements in Table 2. This expert roundtable series illustrates that there is considerable variation in the use of HRF worldwide – from being widely used in some countries to not yet being available in others. Key takeaways are shown in Box 3. In addition to regional differences, discrepancies exist between specialists and non-specialists in most cases, depending on the country. This finding further emphasizes the need for efforts targeting all HCPs prescribing HRF. It also confirms the need for peer-to-peer education and advocacy, supplemented by clinical data. It will be critical to align fully based on published research and the publication of future international CMA guidelines. Agreement was reached that HRF, where available, can be recommended as a possible first-line alternative to cow's milk-based eHF or AAF in the dietary management of CMA.

Box 3 **Key takeaways**

**Table:** 

• There was consensus that a comprehensive diagnosis and appropriate diagnostic procedures can help to ensure all hypoallergenic formulas, like HRF, are appropriately prescribed, but there is room for improvement in this area.
• A distinction needs to be made illustrating the key differentiating factors between currently prescribed hypoallergenic formulas (eg, AAF, eHF, and HRF), particularly in countries where a variety of hypoallergenic formulas are readily available.
• While specialists follow international CMA guidelines, non-specialists are more likely to use local guidelines and/or guidance from professional associations as it more practical. It is critical that international CMA guidelines provide updated information on HRF so that this information can be disseminated and translated into local guidelines.
• Peer-to-peer support, particularly confidence among physicians who have used HRF before, along with positive real-life case studies, is critical to drive widespread acceptance of any new hypoallergenic formula such as HRF.
• HRF use has been limited by geographical availability and lack of awareness among HCPs.
• Once HRF becomes more readily available and first-line prescribers become more familiar with its benefits, there is potential for uptake of HRF as a first-line CMA dietary management option.

CMA, cow's milk allergy; HCP, healthcare professional; HRF, hydrolyzed rice formulas.

Alt-text: Box 3

## Abbreviations

AAAAI; American Academy of Allergy, Asthma, and Immunology, AAF; amino acid-based formula, ASCIA; Australian Society of Clinical Immunology and Allergy, BSACI; British Society for Allergy & Clinical Immunology, CMA; cow's milk allergy, DRACMA; Diagnosis and Rationale for Action against Cow's Milk Allergy, EAACI; European Academy of Allergy and Clinical Immunology, eHF; extensively hydrolyzed formula, ESPGHAN; European Society for Paediatric Gastroenterology Hepatology and Nutrition, FPIES; Acute food protein-induced enterocolitis syndrome, HCP; healthcare professional, HRF; hydrolyzed rice formula, IgE; immunoglobulin E, (i)MAP; (International) Milk Allergy in Primary Care, JSA; Japanese Society of Allergology, JSPACI; Japanese Society of Pediatric Allergy and Clinical Immunology, NIAID; National Institute of Allergy and Infectious Diseases, NICE; National Institute for Health and Care Excellence, SAEPAP; Spanish Association of Paediatric Primary Care, SEGHNP; Spanish Society of Paediatric Gastroenterology, Hepatology, and Nutrition, SEICAP; Spanish Society of Paediatric Clinical Immunology, Allergy, and Asthma, SEPEAP; Spanish Society of Extra-hospital Paediatrics and Primary Health Care, SF; soy formula, UK; United Kingdom, US; United States, WHO; World Health Organization

## Funding

The consensus statements and supporting evidence presented in this paper were discussed and formulated at a virtual expert roundtable series. All authors received support from Abbott to attend the meetings. The views expressed in this paper are purely those of the authors without any influence from Abbott. Publication fees were paid by Abbott.

## Availability of data and materials

Not applicable.

## Author contributions

This consensus was led by RM, who contributed significantly to the review and finalization of the manuscript. All other authors contributed to the development and finalization of the consensus statements and manuscript. All authors have read and agreed to the published version of the manuscript.

## Ethics approval and consent to participate

Not applicable.

## Consent for publication

The authors have reviewed the final version and consent to its publication in WAO Journal.

## Declaration of competing interest

Rosan Meyer has participated as a clinical investigator, and/or advisory board member, and/or consultant, and/or speaker for Abbott, Mead Johnson, Nestlé Nutrition Institute, Nutricia/Danone. Christophe Dupont has participated as a clinical investigator and/or advisory board member and/or consultant and/or speaker for Abbott, Nestle France, Nestle Health Institute, Nestle Waters, Novalac, Nutricia, Sodilac, and is shareholder—Table cofounder of DBV Technologies. Alessandro Fiocchi has participated as a clinical investigator and/or advisory board member and/or consultant and/or speaker for Abbott SA, Immune, Danone SA and serves as Head of the Food Allergy Committee for the World Allergy Organization. Helen Howells has participated as a clinical investigator and/or advisory board member and/or consultant and/or speaker for Abbott, Danone, Mead Johnson, Nestle and Nutricia, and is trustee for the Anaphylaxis Campaign. Raanan Shamir has participated as a clinical investigator and/or advisory board member and/or consultant and/or speaker for Abbott, Else, Nestle, Nutricia, and is shareholder of Else and NGS. Carina Venter has participated as a clinical investigator and/or advisory board member and/or consultant and/or speaker for Abbott, Nestle Nutrition Institute, Reckitt and Sifter. She has held leadership roles in the following medical societies: AAAAI, EACCI, INDANA. Josefa Barrio-Torres declares no conflict of interest.
